# Marasmus and Diphtheria

**Published:** 1913-06-21

**Authors:** H. B. Morgan

**Affiliations:** Junior Assistant Medical Officer, Park Hospital for Children, Hither Green, S.E.


### MARASMUS AND DIPHTHERIA.

H. B. MORGAN, Junior Assistant Medical Officer, Park Hospital for Children, Hither Green, S.E.

The unscientific term marasmus, which literally means a wasting, has been commonly stated in death certificates as a cause of death. Latterly it has been fashionable to substitute the indefinite terms atrophy or debility in the same class of case. The condition marasmus, however, is not a disease, but only a symptom, of toxæmia. It has long been recognised that many cases classed as marasmus are really cases of congenital syphilis or infantile tuberculosis. In some cases the diagnosis can only be made *post-mortem*. Excluding these causes there still remains a certain varying proportion of cases in which a most careful autopsy, with subsequent histological examination and even perhaps animal inoculation, reveals no grounds for a diagnosis of either syphilis or tuberculosis. From a careful clinical study of the great number of marantic children treated in the wards under my control at the Park Hospital for Children, the conclusion is irresistible that the marantic condition results purely from a chronic toxæmia. Many of the children had one or other of the infectious diseases, others had not. The condition of some dated from an acute illness, others slowly and gradually wasted away. Was the marasmus a sequel to a severe attack of an acute fever, a protracted convalescence, or was it essentially a slow, chronic, insidious poisoning? Many observations favoured the latter view, and investigations were undertaken, based on this thesis, for the purpose of testing the accuracy of the view or the reverse. A negative result was considered likely to prove as valuable as a positive one.

### Diphtheritic Toxonæmia.

Investigation early demonstrated that the part played by the diphtheria bacillus in the causation of marasmus was not sufficiently realised or emphasised. In some cases of marasmus the condition is really one of chronic diphtheritic *toxonæmia*. There were no manifestations of diphtheria as clinically recognised. The frequency, however, with which marantic children suddenly developed diphtheria was striking. These were sporadic cases, and gave rise to no further infection in the ward. Nasal discharges, watery, mucous, or purulent, were present more or less in about 65 per cent. of these children. In the rest there was no evidence of any rhinitis or rhinorrhœa. The throat and faucial condition appeared usually quite normal.

The term marasmus is very loosely applied; but the typical marantic child's weight is much below the normal; the degree of emaciation varies; pallor and anæmia are usually striking, the actual degree depending on the duration of the marantic condition and the reaction of the infantile system against the toxone. The anæmia is a secondary one. The skin is loose, pale, and often puckered. The child is irritable and restless, lies moaning and crying itself to exhaustion point almost all day. His appetite is good, and he is apparently never satisfied even when filled to nauseation with properly diluted milk feeds. He reacts very indifferently to thermogenetic or fever-producing poisons. Though he is obviously ill, a condition, which in a healthy infant would cause marked pyrexia, barely accounts for a rise of a degree in his case. This is not so in every case, but it is frequently seen. The conditions of the stools vary: they may be quite normal, or relaxed and with a varying depth of green.

In a few cases a culture on Loeffler's blood serum from a swab of the nose or nasal discharge would show the presence of colonies of the diphtheria bacillus. More frequently cultures from swabs of each nostril and the fauces have to be taken on many occasions (5 to 8 times) before a positive result is obtained. The frequency with which the presence of the diphtheria bacillus is proved in these cases of marasmus depends to a large extent on the assiduity and persistence of the search. A few stray, suspicious bacilli may occasionally be found in smears from primary cultures; in such cases the search should be continued with redoubled energy and swabs taken frequently. Smears from such cultures should always be stained by Neisser's or Cobbett's method. The difficulties of the search are enormous. Even, however, in cases of clinical diphtheria, the bacillus on cultural examination is not found in about 21 per cent. of cases; the percentage in cases in which there are absolutely no signs suggestive of diphtheria (except an occasional rhinorrhœa) would proportionately be very much less.

### The Production of a Vicious Circle.

The theory is that in such cases of marasmus the diphtheria bacillus produces a larger amount of toxone than usual; or the toxin for some unknown reason is converted into toxone. The diphtheria bacillus may be present but unable to obtain a stronger hold. The vitality of such children is low; their tissues may lack some constituent necessary for a virulent growth and spread. Membrane is never or seldom seen in these cases of chronic diphtheria in marasmus. A very small factory of diphtheria bacillus is present in the nasal mucous membrane or post-nasal area, producing a very small and limited, but constant, amount of diphtheria toxin and toxone. Against this poisonous exotoxone the child's tissues are incessantly engaged. While not in sufficient amount to produce acute and prominent clinical manifestations the exotoxone has the effect of ultimately producing symptoms of cachexia, slow, progressive loss of weight and emaciation, and, concomitantly, pallor and anæmia. The other symptoms are sequelæ of this result—and so a vicious circle of symptomatology is apt to be produced, all depending on this unobtrusive microbic influence.

It is well known that membranous rhinitis in adults, now recognised as due to the diphtheria bacillus of a virulent nature, is often attended with few general symptoms, and practically without risk to life. The adult is better fitted to withstand the effects of the toxonæmia than the infant.

Recently Colombet, a French writer, in a thesis on the sequelæ of unrecognised diphtheria in the adult, recognised four groups: (1) Nervous, with or without paralysis; (2) cutaneous; (3) cardio-renal; and (4) diphtheritic cachexia—viz. extreme weakness, wasting, anæmia (due to diminution in number of red cells), palpitation, and tachycardia.

### Classification of Marasmus.

Marasmus in the infant seems easy of classification into the fourth group. As the condition is due to an exotoxonæmia the paucity of pathological changes *post-mortem* is easily understood. The condition is due to long-continued absorption in small doses of a certain poison elaborated and manufactured by the bacilli. The production of a chronic diphtheritic condition in susceptible animals seems confirmatory of these theses. Clinical records and investigations in four wards of marantic children during the past eight months also favour this view. Investigations are being continued—coprological, serological, hæmatological—to test the accuracy of this theory and for recording a few facts as to the bacteriology of the condition. Statistics and records are being accumulated. The bacilli are being tested for the carbohydrate reactions of the Klebs-Lœffler's bacillus, and so far with confirmatory results. The virulence of the organisms isolated varies. On a few occasions the bacilli were only isolated or found some hours after death, as the culture medium was inoculated twelve to sixteen hours before death. Previous search in such cases had been negative. This is interesting, as two months ago—some months after these investigations were in progress—a case of bacillary septicæmia due to this organism was described in the *American Journal of Children's Diseases*. The bacillus was found in some organs and in the blood after death.

The literature on the subject is very scanty. Standard works on diphtheria have no references to this relationship between diphtheria and marasmus. The case reported above was regarded as a rarity. And the search for references has so far had little result.

### The Sociological Point of View.

This question is of great sociological interest from the point of view of infantile mortality. If the thesis ultimately proves to be scientifically accurate, it is evident that the ubiquitous diphtheria bacillus in its relation to the home conditions and environment of these weaklings will have to be further studied. For frequently this diphtheritic toxonæmia is superimposed on either a syphilitic or tubercular condition. In a case of congenital syphilis under my care the condition was unsuspected till the child suddenly developed pneumonia. An accompanying nasal discharge proved on examination to contain Klebs-Lœffler bacilli in pure culture.

Many observers have confirmed the observation that the diphtheria bacillus is present in a certain proportion of apparently perfectly healthy people and in a "not inconsiderable proportion" of school children. These constitute the vexed problem of "diphtheria carriers." Considerable discussion has taken place as to their virulent or non-virulent condition. In considering the relation of this bacillus to marasmus it must be remembered that young tissue is very susceptible to bacillary infection, and a bacillus which may prove merely saprophytic to a school child of moderate physique and colour may be quite harmful to an infant reared under economic conditions the reverse of favourable to its development. Even this modified bacillus may be capable of producing this toxonæmia and marasmus in a susceptible infant.

Toxone is an original product of the diphtheria bacillus. This hypothesis of marasmus necessitates the view that according to Ehrlich's side chain theory of immunity the toxone on absorption stimulates the production of antitoxin (antitoxone), but never in sufficient amount to neutralise completely the whole of the toxone. The free toxone attaches itself by its "toxophore" and "haptophore" groups to the "toxophile" and "receptor" groups respectively of the cell protoplasm, and by this union or combination exerts its deleterious effect. By the slow, long-continued action of this combination a marantic condition with its train of sequels is produced. The toxone is always being manufactured by this "factory" in the nasal area and in sufficient amount to preponderate over the amount of antitoxone produced by the body cells.

In a further article it is hoped to review a series of cases with clinical records in illustration and in support of this thesis.

